# The Behaviour and Productivity of Mid-Lactation Dairy Cows Provided Daily Pasture Allowance over 2 or 7 Intensively Grazed Strips

**DOI:** 10.3390/ani8070115

**Published:** 2018-07-11

**Authors:** Megan Verdon, Richard Rawnsley, Pieter Raedts, Mark Freeman

**Affiliations:** Tasmanian Institute of Agriculture, College of Sciences and Engineering, University of Tasmania, Tasmania 7320, Australia; richard.rawnsley@utas.edu.au (R.R.); peter.raedts@utas.edu.au (P.R.); mark.freeman@utas.edu.au (M.F.)

**Keywords:** dairy cow, grazing management, moomonitor^+^, pastoral dairy production, strip-grazing

## Abstract

**Simple Summary:**

Recent technological advances will soon allow pastoral dairy farmers to manage their cattle using increasingly intense and complex grazing regimes. Ensuring there is merit in the implementation of more intense grazing regimes will minimise the potential misuse of this technology and the associated consequences of misuse for animal welfare and productivity. Two groups of dairy cattle were provided with the same amount of fresh pasture over either 2 or 7 feeds per day. Cows that received pasture over 7 feeds produced less milk and spent less time ruminating, but more time resting, per day. However, feeding frequency did not affect the time cows spent feeding or estimated pasture consumption. Increasing the frequency of feeding restricts the amount of pasture available to cattle at any one time. In response, cattle adjust their ingestive behaviour (e.g., adopt high intake rates) which may negatively impact digestive processes. The success of more intensive pastoral dairy production requires grazing regimes that support the natural ingestive, digestive, and social behaviours of cattle, rather than necessitating cattle to substantially alter their behaviour in accordance with the grazing regime imposed upon them.

**Abstract:**

Research into the effects of intense grazing regimes on cattle behaviour and productivity will support the ethical intensification of pastoral dairy production. Two treatments were applied to two herds of 30 mid-lactation cows over 28 days. Cows were offered an estimated 12 kg DM/cow (above 5 cm from ground level) of irrigated pasture per day. The control herd received their daily pasture allocation in two equal grazings while the experimental herd received theirs over seven smaller grazings. Backgrazing beyond the current allocation (morning or afternoon) was prevented. Individual records were taken daily for milk production and behaviour (MooMonitor^+^). Milk composition, energy corrected milk (ECM), and live weight were recorded weekly. Feeding mid-lactation dairy cows over seven smaller grazing allocations reduced the time cows spent ruminating (*p* < 0.001), milk yield (*p* < 0.001), and ECM (*p* < 0.05). However, milk composition, live weight, time feeding, and pasture consumption were not affected by feeding frequency (*p* > 0.05). Cattle may have adapted their ingestive behaviour in response to the more intensive strip-grazing regime utilised in this study, with negative consequences for digestive processes and consequently milk production. Intense grazing regimes need to support the ingestive, digestive, and social behaviours of cattle.

## 1. Introduction

The intensification of animal production is often associated with long periods of confinement and indoor housing [1], rather than with outdoor pastoral production systems. Although the Australian dairy industry has intensified considerably over the past 3 decades (i.e., by increasing stocking rate and the proportion of supplementary grain provided), the key profit driver of dairy production in Australia continues to be home grown feed consumption [2,3]. Intensive grazing regimes, such as the provision of pasture in single or half day allocations (i.e., strip-grazing), are commonly utilised by pastoral dairy farmers. The restricted availability of pasture and higher stocking densities associated with intensive grazing systems enable a more consistent, controlled and efficient utilisation of pasture [4,5], which contributes to a higher forage output per hectare (both quantity and quality, e.g., [6,7]).

Increasingly intense grazing regimes are likely to underpin the continued intensification of animal production in pasture-based systems [8]. At present, the implementation of more intensive strip-grazing systems is limited by costs associated with increased labour and fencing requirements [9]. Virtual fencing (or virtual herding) technology has successfully contained cattle using sensory cues (typically an audio cue that the animal has been trained to associate with a pending electrical stimulus unless it modifies its behaviour) delivered via collars, rather than physical boundaries [10,11]. By allowing flexible spatiotemporal movement of livestock in real time [12], virtual fencing technology may remove some of the barriers currently preventing the implementation of complex and more intense grazing regimes in pastoral dairy systems. In order to minimise the potential misuse of virtual fencing technology, and the associated consequences of misuse for animal welfare and productivity, it is essential that merit in the application of the technology is demonstrated prior to its implementation.

A more intensive version of strip-grazing, characterised by the provision of fresh pasture to dairy cattle in frequent yet small quantities throughout the day, is one example of a grazing regime which may be made practical by virtual fencing technology. Increasing the frequency of pasture allocation could improve pasture utilisation by reducing damage to palatable herbage caused by fouling or treading [9]. A more consistent distribution of feeding time throughout the day may positively affect the efficiency with which milk is synthesised by providing a more consistent supply of metabolites [13]. While Dalley and colleagues [13] reported a 1 L reduction in milk yield when the frequency of pasture allocation for 100% grazed cows increased from once to six times per day, the authors hypothesised this was due to the allocation of pasture during the night increasing the consumption of herbage with lower soluble carbohydrate levels.

The present experiment examined the effect of providing the daily pasture allowance of mid-lactation dairy cows over seven smaller allocations, focused during day light hours, on dairy cow behaviour, and productivity. By increasing our understanding of cattle response to a simple, yet more intense strip-grazing regime, this experiment aimed to produce scientific knowledge that (1) supports the ethical intensification of pastoral dairy production systems; and (2) provides a foundation upon which more complex intensive grazing regimes can be developed. The hypothesis being tested was that the frequent presentation of fresh pasture would increase the time cows spent grazing, pasture consumption, and milk yield.

## 2. Materials and Methods

### 2.1. Ethical Statement

All animal procedures were conducted with institutional ethical approval (University of Tasmania Animal Ethics Committee, A0016234) prior to the start of the experimentation.

### 2.2. Animals and Housing

This experiment was conducted over 4 weeks in summer at the Tasmanian Institute of Agriculture Dairy Research Facility (TDRF) at Elliott, NW Tasmania (−41.093°, 145.780°, 155 m a.s.l.). The mean maximum and minimum daily ambient temperatures (°C) for the period of experimentation were 19.3 and 10.4, respectively. The milking herd at TDRF consists of 360 spring calving cows that rotationally graze approximately 112 hectares of pasture (40 ha irrigated, 72 ha rainfed). The herd is comprised of approximately 30% Holstein Friesian animals with the remainder primarily being cross-bred Friesian × Jersey, with fewer numbers of Friesian × Aussie Red. Cows are milked twice per day (approx. 0530 and 1500) in a 20 aside swing-over herringbone dairy with automatic cup removers, and supplementary concentrate provided in the dairy (2–7 kg/cow per day, 88% DM).

Sixty mid-lactation multiparous (parity range 2–7) dairy cows (*Bos taurus*) from the TDRF milking herd were utilised in this experiment. Over the 28 day experimental period, selected cows grazed eight irrigated paddocks (range 2–5 days grazing per paddock), with one paddock being grazed twice (26 days between rotations). The average (±s.d.) paddock size was 1.4 ± 0.32 ha. Cows were offered an estimated 12 kg pasture DM/cow (6 kg DM/cow after the morning and the afternoon milking) and 4 kg of supplementary concentrate (2 kg/cow during the morning and afternoon milking, 88% DM) per day. Pasture allowances were based on herbage mass estimates measured using a rising plate meter (Farmworks Systems, Feilding, New Zealand) that utilised the following site specific calibrated equation:

y = 240x + 500(1)

where y = estimated pasture biomass in kg DM/ha, and x = number of 1 cm in compressed pasture height. This equation was derived from published [14] and unpublished research conducted on irrigated perennial ryegrass swards in summer taken to ground level at the same site as the present experiment. Importantly, the same quantity of pasture was estimated and offered to all cows using the same rising plate meter and equation. One water trough per 15 cows provided water ad libitum.

### 2.3. Experimental Design

The 60 selected cows were allocated to one of four groups (15 cows per group) that were balanced for breed, age (mean ± s.d.; 5.56 ± 1.61 years), live weight (mean ± s.d.; 532 ± 44 kg), days since calving (mean ± s.d.; 135 ± 22.5 days), and milk production (mean ± s.d.; 22.3 ± 3.1 L/cow per day). These four groups were combined to produce two herds of 30 animals, and two treatments that differed in feeding frequency were applied to these herds. For all cows, grazing commenced after each of the two milkings per day (cows removed from paddock for milking at approx. 0700 and 1430 h). Cows in the control herd received their daily pasture allowance in two equal grazings per day (approximately 0830 and 1530 h; 2 allocations per day, labelled 2A). For cows in the treatment herd, the morning pasture allocation was provided in 4 equal strips (approx. 0830, 1000, 1130, and 1300 h) and the afternoon pasture allocation in 3 equal strips (approx. 1530, 1830, and 0530 h). Thus, treatment cows received their daily pasture allowance in seven grazings per day (7 allocations per day, labelled 7A). For convenience, in the remainder of this paper, the provision of a strip of pasture to cows from the treatment herd is described as belonging to the ‘AM’ or ‘PM’ pasture allocation, depending on the time of day that the pasture was measured and allocated, rather than when each portion of the pasture was provided.

This experiment was a high-order crossover study that incorporated Balaam’s design, which is characterised by the application of two treatments (e.g., control treatment X and experimental treatment Y) over two periods in one of four possible sequences: XY (control-treatment), YX (treatment-control), XX (control-control), and YY (treatment-treatment). This hybrid of a crossover design and a parallel design allows for unbiased estimations of differences in the presence of carryover effects [15]. In the present experiment, 2A and 7A treatments were applied to herds of 30 cows for an initial period of 14 days. This included a 3 day adjustment period during which no data was collected, followed by 11 days of data collection. An adjustment period of 3 days was selected on the basis that milk yields normalised after this period. After 14 days, one group of cows from the 7A herd (*n* = 15 cows) and one group of cows from the 2A herd (*n* = 15 cows) crossed treatments. The other two groups of cows remained in the experimental or control treatments. The treatments were then applied to the new herds of 30 cows for a second period of 14 days. Again, cows had a 3 day adjustment period followed by 11 days of observation. Thus, two treatments were applied to two herds of 30 cows over two periods, with the composition of the 2A and 7A herds being different from days 1 to 14 compared to days 15 to 28.

Paddocks were evenly divided into two sub-paddocks using electrified galvanised steel fencing wire, which allowed the two experimental herds to graze the same paddock side by side. The sub-paddock being grazed by each herd alternated daily. The following milking and grazing procedures were consistent with conventional practise. Grazing commenced after each of the two milkings per day. Backgrazing beyond the current allocation (morning or afternoon) was prevented using electric fences. This also allowed a more reliable estimate of pasture consumption (see measures recorded) per grazing to be calculated. It took approximately 45 to 60 min to collect, milk, and draft the 60 cows. The 2A herd trickled back to their paddocks after milking (as per conventional practise), but 7A cows were drafted upon exit from the dairy and moved to their paddock as a herd. This ensured all cows in the experimental treatment had equal access to fresh pasture in the first grazing period post-milking.

### 2.4. Measures Recorded

#### 2.4.1. Milk Yield

Milk yield of each cow, at each milking (morning and afternoon), was recorded using a DeLaval Alpro milk metering system (DeLaval International AB, Tumba, Sweden) at the day prior to the commencement of the experiment (day −1) and at each day of the experiment. As milk secretion is a continuous process in lactating cows [16], the effects of the AM grazing allocation will likely be associated with milk production in the afternoon of that same day, while the effects of the afternoon grazing allocation will not be evident until the morning milk production of the following day. Thus, a “day” of milk production refers to the afternoon milking on one day plus the morning milking on the following day (e.g., day 1 milk yield = milk yield PM day 1 + milk yield AM day 2).

#### 2.4.2. Milk Composition

Milk composition samples were collected at both the morning and afternoon milking at day −1, and weekly thereafter (days 7, 14, 21, and 28). Milk samples were assayed for fat and protein concentrations (g/kg), and for somatic cell count (SCC) using a Bentley B2000 Infrared Milk Analyser (Bentley Instruments Inc., Chaska, MN, USA) by TasHerd Pty Ltd. (Haspen, Tasmania, Australia). The following milk composition variables were then calculated for each cow: (1) kilograms of fat and protein per sample day; (2) percent of fat and protein per sample day; (3) the average SCC (cells/mL) per milking and sample day; and (4) the fat and protein corrected milk yield (FPCM). The FPCM provides an indication of the amount of milk produced adjusted to a standard of 4.00% fat and 3.40% total protein, and was calculated using the following formula [17]:

FPCM (kg) = milk yield (kg) × ((0.383 × % fat + 0.242 × % protein + 0.7832)/3.140)(2)

#### 2.4.3. Live Weight

Cow live weight was measured twice daily using automatic in race walk over scales (DeLaval AWS100 automatic weighing system) as animals exited the dairy. The AWS100 software utilises an algorithm that discounts recorded weights that differ greatly from the 7 day mean weights for individual animals (e.g., instances when more than one cow is on the platform at any one time). Thus, the 7 day average weight data provides a more accurate representation of cow live weight and live weight changes.

#### 2.4.4. Time Feeding, Ruminating and Resting

Each cow was fitted with a neck-collar mounted activity monitoring system (MooMonitor^+^, Dairymaster Inc., Kerney, Ireland) at day −1. The commercially available MooMonitor^+^ uses a 3-dimensional accelerometer to determine cow movement and head direction, and, based on proprietary algorithms, distinguish between feeding, resting, and ruminating behaviours [18]. Using the MooMonitor^+^, the present experiment continuously monitored the time cows spent feeding, resting, and ruminating, and used this data to calculate cow behaviour per day and per hour. For the purpose of this study, a “behavioural day” is defined at the 24 h starting from 0700, when cows were transferred from paddock to the dairy.

#### 2.4.5. Pasture Consumption

Estimated pasture intake (kg DM/cow) for the herd was calculated from pre- and post-biomass estimates using the rising plate meter (for details, see Section 2.2). As per Pembleton et al. [14], the same calibration equation was utilised for pre- and post-biomass estimates. At least one-hundred readings were taken in each treatment area before and after grazing. For both the AM and PM grazing, estimated pasture intake was calculated as the difference between the pre- and post-grazing herbage mass, averaged per cow in the herd. The estimated daily pasture intake was then calculated as the sum of the estimated pasture intake at the AM and PM grazing (kg DM/cow day).

### 2.5. Statistical Analysis

Two cows (one from each treatment) were removed within 24 h of the trial commencing due to either lameness or mastitis and were replaced with animals that were comparable in weight, parity, days in milk, and milk yield. No data from days −1 or 1 were available for these replacement cows. Two cows were temporarily removed during the experimental period to be treated for mastitis (1 × 2A cow from days 19 to 23 and 1 × 7A cow from days 26 to 28). Pasture allocations were adjusted in their absence. Extreme values (greater than 3 × SEM) for variables relating to milk composition, milk production, and behaviour, and that were not characteristic of the individual cow (based on previous and following 3 days of data), were assessed to be technical errors and so removed as outliers for that specific day (3% of milk composition data, 1% of milk production data, and 0.2% of behaviour data). Due to a technical malfunction no data on milk production were obtained for days 18 and 19 (9% of milk production data).

Data collected from animal-based measures has a hierarchical structure in that individual cows were nested within herds of 30 animals. Thus, the possible nonindependence of observational units in our experiment cannot be eliminated. This limitation could not be overcome practically with a different experimental design [19]. There is ongoing controversy as to whether individual animals may be accepted as independent replicates [19,20,21,22,23,24,25]. In the present experiment, the effects of any interdependence between cows on the estimation of treatment differences is likely negligible because (1) the crossover design imposed varying social conditions on individuals; and (2) behavioural durations are less likely than frequencies to be influenced by social effects [26,27]. Further, multi-level modelling (i.e., hierarchical linear models, or nesting) are able to assess correlations within groups while also quantifying variability between groups [20,28]. Such statistical models have been advocated [20,22] and utilised [29,30,31] to overcome many perceived issues associated with statistical nonindependence, and are therefore used by the present experiment. Nonetheless, care should be taken when ascribing causation to treatment effects in the present experiment.

All statistical analyses were carried out using either general linear mixed model (LMM) or generalized linear mixed model (GLMM) function of the SPSS statistical software package (SPSS 22.0, SPSS Inc., Chicago, IL, USA). Normality was assessed prior to statistical analysis using visual methods (quantile-quantile plots and histograms). Data that were not normally distributed were transformed prior to analysis, so that the residual variation was homogenous between treatments within periods. Specific transformations are indicated in the results. When an interaction was not significant it was removed from the model so that significant effects could be more reliably interpreted. Where there were significant main or interactive effects (*p* ≤ 0.05) the LSD test determined where least square means differed. Data are presented as least square means and pooled standard errors. For the transformed variables, transformed least square means and pooled standard errors are presented with back transformed means presented in parenthesis.

The statistical models used are detailed in the following paragraphs and summarised in Table 1. A LMM was used to ensure that milk composition at day −1 was balanced across the four groups of 15 cows. Data for fat and protein (kilograms and percent, per day), FPCM (kg per day), SCC (average per day), and live weight (averaged over the previous 7 days) at days 7, 14, 21, and 28 were analysed using LMM. Each model accounted for repeated observations of the same cow (nested within period) over sample days with a first order auto-regressive correlation structure. The term “prior treatment” was introduced to assess crossover effects. In period 1, cows did not have a prior treatment (set to “none” for all cows). In period 2, “prior treatment” refers to the treatment the cow was in for period 1. The fixed effects of treatment, sample day (within period), a treatment × sample day (within period) interaction, and prior treatment were fitted to the model. Milk composition and live weight (averaged over the previous 7 days) variables obtained at day −1 were fitted to relevant models as covariates.

The effects of treatment on daily milk yield and the time cows spent ruminating, resting, and feeding per day were assessed using LMM’s. Each model fitted treatment, prior treatment, and their interaction as fixed effects, while paddock was included in the model as a random blocking factor. Milk production at day −1 was included in the analysis of milk yield as a covariate. Behaviour per hour was rounded to the nearest minute to represent a count of occurrences (i.e., number of minutes performing behaviour) in a fixed period of time (i.e., 60 min). GLMMs with an underlying Poisson distribution and log link function were then developed to assess the effects of treatment on behaviour per hour. A negative binomial distribution was fitted when behaviour data per hour were over dispersed. To simplify the analysis, separate models were developed for each hour within a day. The model specified treatment as a fixed effect, while paddock was controlled for as a random effect. Observations on the same individuals were repeated longitudinally over days, so each model accounted for repeated observations of the same cow (within period) over day with a first order auto-regressive correlation structure.

To explore the effects of treatment on the relationships between cow behaviour (time feeding, ruminating, and resting per day), milk production (L milk yield per day), and characteristics of the individual cow (age, weight), partial correlations that controlled for day, paddock, and period were conducted for each treatment.

The effects of grazing treatment on daily pasture consumption were analysed using LMM. The subject of analysis was the grazing day nested within paddock (within period). Treatment period and their 2-way interaction were fitted to each model as fixed effects. Characteristics of the pre-grazed sward can effect bite mass and intake rate (reviewed by [32]), so pre-grazing pasture biomass was included in these models as a covariate.

## 3. Results

### 3.1. Milk Production

**Figure 1:**
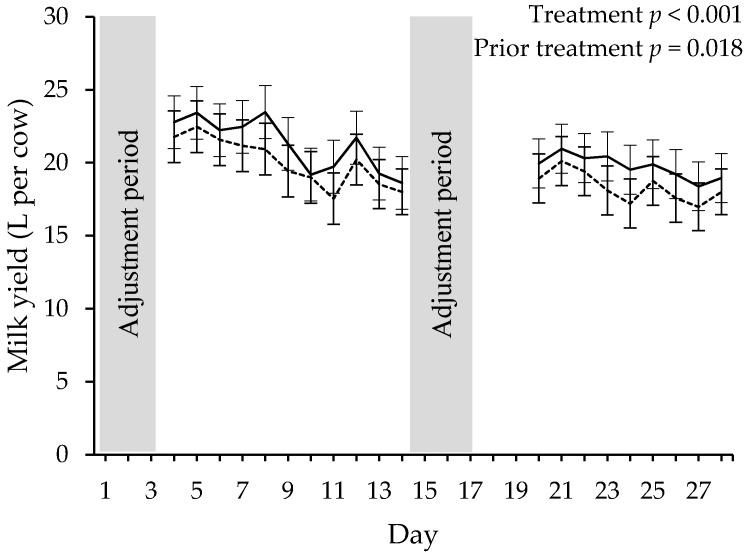
Changes in milk yield (litres per cow) for cows fed daily pasture allocation in 2 feeds per day (solid line) or 7 feeds per day (dotted line) over 11 days prior to (days 4–14) and 9 days following (days 20–28) an incomplete treatment cross-over at day 15. No data were collected in the adjustment periods (days 1–3 and 15–17). Due to a technical malfunction, no milk yield data were recorded at days 18 and 19. Least square means ± 95% confidence interval, calculated using linear mixed models, are presented.

Cows offered fresh pasture twice per day produced more litres of milk than those fed fresh pasture 7 times per day (19.4 ± 0.68 vs. 18.2 ± 0.68 L per cow and day; F_1,111_ = 27.5, *p* < 0.001; Figure 1). Milk yield for all cows was lower in period 2 compared to period 1 (main effect of prior treatment F_2,187_ = 59.7, *p* < 0.001; Figure 1). In period 1, cows produced 20.9 ± 0.68 L per day. In period 2, cows from the 7A prior treatment produced 17.4 ± 0.71 L per day whereas cows from the 2A prior treatment produced 18.1 ± 0.71 L per day.

### 3.2. Milk Composition

The four groups of 15 cows did not differ (*p* > 0.05) in mean milk fat and protein concentrations (means ± s.d., respectively, were 51 ± 8.5 and 32 ± 2.9 g/kg), measured at the day prior to the application of treatments.

There was no effect of treatment on the percent of fat or protein in daily milk production (*p* > 0.05, Table 2). However, the kilograms of fat (F_1,140_ = 3.91, *p* = 0.05) and FPCM (F_1,105_ = 5.04, *p* < 0.05) produced per day was lower, and there was a tendency for the kilograms of protein produced per day to be lower (F_1,108_ = 3.34, *p* = 0.07), in the 7A compared to the 2A cows (Table 2). The kilograms of fat (F_2,135_ = 47.4, *p* < 0.001), protein (F_2,56.3_ = 63.2, *p* < 0.001), and FPCM (F_2,110_ = 79.2, *p* < 0.001) produced each day was lower at the second sample day in a period than in the first (see Day 7 vs. Day 14 and Day 21 vs. Day 28, Table 2), irrespective of experimental treatment. There were no effects of prior treatment (i.e., crossover effects) for any milk composition variables (*p* > 0.05, Table 2). There was a tendency for SCC to be lower in the 2A cows (F_1,247_ = 3.00, *p* < 0.1), while SCC increased over days for both treatments (F_2,247_ = 7.46, *p* < 0.001; Table 2). There was no effect of sample day on the percent of fat or protein in milk (*p* > 0.05, Table 2).

### 3.3. Behaviour

Probability values and least square means for the effects of treatment and prior treatment on the time cows spent feeding, resting, and ruminating per day are presented in Table 3. Cows that were fed pasture 7 times per day (i.e., 7A cows) spent more time resting (F_1,114_ = 7.2, *p* < 0.05), and less time ruminating (F_1,114_ = 31.3, *p* < 0.001) than cows allocated pasture twice per day (i.e., 2A cows). However, there was no effect of treatment on the time cows spent feeding (F_1,114_ = 0.005, *p* > 0.05). There was a significant effect of prior treatment on the behaviour variables of feeding (F_2,169_ = 3.11, *p* < 0.05) and ruminating (F_2,183_ = 26.1, *p* < 0.001), but not resting (F_2,166_ = 1.73, *p* > 0.05). The crossover did not affect the time cows spent feeding and ruminating. However, cows that were assigned to the 2A treatment for period 1 spent more time feeding in period 2, regardless of treatment in period 2, and all cows spent less time ruminating in period 2 than period 1 (Table 3).

**Figure 2:**
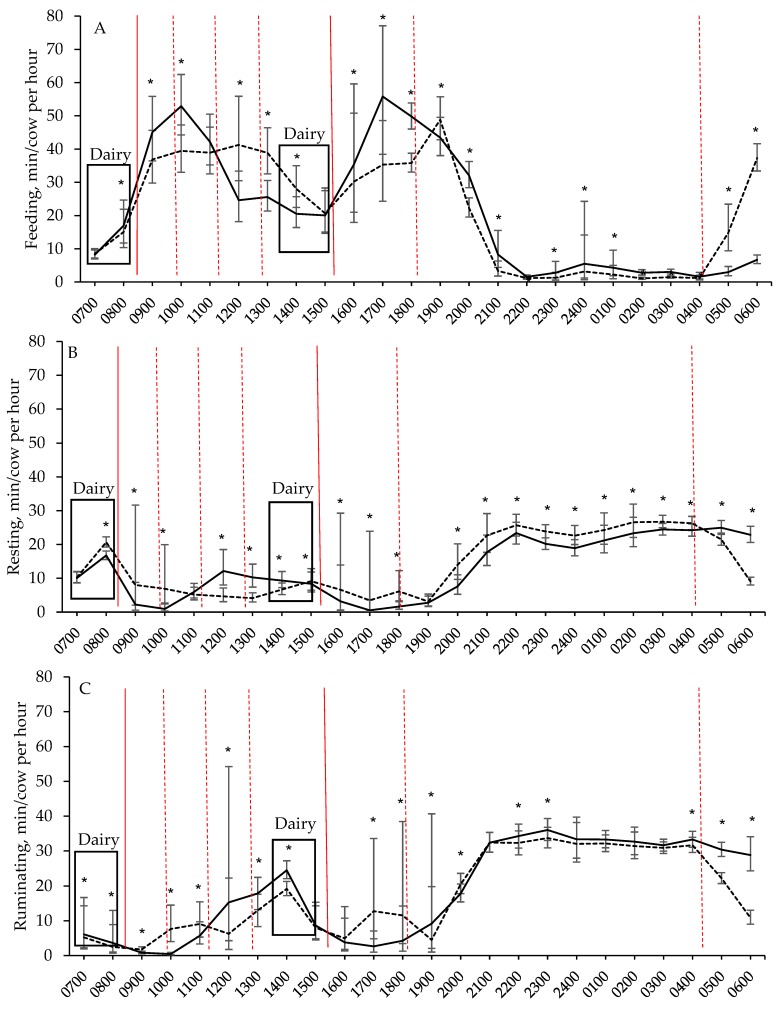
The effects of providing cows their daily pasture allocation over 2 feeds per day (solid line) or 7 feeds per day (dotted line) on time (*y*-axis) spent (**A**) feeding, (**B**) resting, and (**C**) ruminating, over 24 h per day (*x*-axis). The approximate times that all cows gained access to fresh pasture are indicated by a solid red reference line, while the approximate times that feeds were provided only to cows fed 7 times per day are indicated by a dotted red reference line. Least square means ± 95% confidence interval, calculated using linear mixed models, are presented. Significant differences between treatments are indicated by * (*p* < 0.05).

When the effects of treatment were analysed for each hour within a day, it was apparent that the behaviour of cows in the two treatments differed more often than not (Figure 2). Time feeding per hour increased when fresh pasture became available for both treatments (Figure 2A). For 2A cows, time feeding per hour peaked approximately 1.5 h after receiving fresh pasture. The decline in feeding behaviour per hour coincided with an increase in time resting per hour (Figure 2B) for 2A cows, and, to a greater extent, time ruminating per hour (Figure 2C). The time 7A cows spent feeding per hour also peaked at approximately 1.5 h after receiving fresh pasture, but to a lower level that persisted for a longer time compared to 2A cows (Figure 2A). Consequently, 7A cows spent less time resting and ruminating per hour, but more time feeding per hour, than 2A cows after the initial morning grazing period (i.e., from 1100 to 1400 h). Further, 2A cows spent more time feeding and less time resting per hour overnight than 7A cows (i.e., 2000 to 0400 h), and there is some indication that 2A cows also spent more time ruminating per hour overnight (see 2200, 2300 and 0400 h; Figure 2C).

### 3.4. Live Weight

The live weight of cows at days 7, 14, 21, and 28 are presented in Table 2. Cow weight was not affected by treatment (F_1,210_ = 0.09, *p* > 0.05) or by prior treatment (F_1,211_ = 2.51, *p* > 0.05). Irrespective of treatment, all cows weighed more at the first sample day in a period than at the second (F_2,116_ = 78.5, *p* < 0.001; Day 7 vs. Day 14 and Day 21 vs. Day 28, Table 2).

### 3.5. Relationships between Behaviour, Milk Production and Cow Characteristics

There were few significant correlations between cow behaviour, productivity, and physical characteristics (Table 4). The correlation coefficient for many of the significant relationships were less than 0.2, indicating that these relationships are negligible [33].

Age was negatively correlated with milk yield for cows in the 2A treatment (*r* = −0.29, *N* = 599, *p* < 0.001), but positively correlated to milk yield for cows in the 7A treatment (*r* = 0.26, *N* = 565, *p* < 0.001). There was no relationship between start weight (i.e., at day −1) and milk yield for cows in the 2A treatment (*r* = 0.04, *N* = 559, *p* = 0.38). By contrast, the start weight of cows in the 7A treatment (i.e., at day −1) was positively correlated with milk yield (*r* = 0.26, *N* = 565, *p* < 0.001).

### 3.6. Pasture Consumption

On average, the estimated pre-grazing herbage mass to ground level was 3332 ± 422 kg DM/ha. There were no interactive effects in the analysis of estimated pasture consumption (*p* > 0.05). Estimated pasture consumed per day was comparable between 2A (10.3 ± 0.17 kg DM/cow day^−1^) and 7A (10.3 ± 0.20 DM/cow day^−1^) treatments (F_1,82_ = 0.01, *p* = 0.92), but both treatments consumed more in period 2 (10.7 ± 0.21 DM/cow day^−1^) than period 1 (9.7 ± 0.09 DM/cow day^−1^; F_1,82_ = 13.3, *p* < 0.001). The estimated mean (±s.d.) post-grazing herbage biomass was 1759 ± 144 kg DM/ha and 1782 ± 141 kg DM/ha for the 7A and 2A treatments, respectively.

## 4. Discussion

The results of the present experiment show that, in comparison to cows that were offered fresh pasture twice per day (i.e., 2A treatment), feeding mid-lactation dairy cows their daily pasture allowance over seven smaller grazing strips per day (i.e., a more intensive strip-grazing, 7A treatment) results in a reduction in milk yield and time spent ruminating, and an increase in time spent resting. However, the more intensive strip-grazing regime utilised in the present study did not affect milk composition, pasture consumption, or the time cows spent feeding per day.

Research by Dalley and colleagues [13] found reduced milk production in dairy cows that were fed fresh pasture six times rather than once per day. However, grazing, ruminating, and resting behaviour (observed at 10 min intervals on 2 days that were one week apart) were not affected by feeding frequency [13]. The authors hypothesised that allocating pasture during the night increased consumption of herbage with a lower soluble carbohydrate level (see [34]), resulting in a reduction in milk yield. The present experiment, which continuously recorded grazing, ruminating, and resting behaviour, did not offer fresh pasture overnight to either treatment. In fact, cows in the 7A treatment of the present study may have had more opportunities to consume pasture of higher soluble carbohydrate concentration than those in the 2A treatment: cows in the 7A treatment were offered 14.3% of their daily pasture allocation at 0530, when soluble carbohydrates are approximately at their lowest levels, but another 14.3% at 1800, when soluble carbohydrates are around peak levels [34]. Of the remaining 70% (approx.) of pasture allocated to 7A cows daily, 60% was offered 1.5 to 4.5 h after 2A cows gained access to fresh pasture.

While cows in the 7A treatment of this experiment were offered the same daily pasture allowance as those in the 2A treatment, the quantity of dry matter available to 7A cows at any one time was temporally restricted by the incremental delivery of pasture. Cattle compensate for restricted pasture availability by increasing bite mass and/or decreasing the time taken to masticate each bite [35]. The delivery of pasture to 7A cows as small but frequent meals may also have delayed feelings of satiation. When herbage availability and accessibility is not a constraint, fasted dairy cows can consume their daily herbage allowance in ~40% or less of the grazing time spent by nonfasted cows [36]. Thus, cows in the 7A treatment of this research may have adjusted their ingestive behaviour in response to temporal restrictions in pasture availability and prolonged feelings of hunger, resulting in multiple periods with high intake rates. The negative consequences of high intake rates on productivity have been reviewed by Gregorini et al. [36]: high intake rates result in poor mastication and improper packing of ingesta, leading to longer retention times of digesta in the rumen, and, thereby, increased nonglucogenic and methanogenic rumen fermentation patterns which reduces energy utilisation for productive purposes.

The frequency, quantity, and timing of pasture allocation in the present experiment may also have disrupted normal grazing and rumination cycles. Firstly, while rumination generally follows ingestion, feeding has priority over rumination whenever the causal factors of the two behaviours are in conflict [37,38]. Gregorini and colleagues [39] found that grazing dairy cows compensate for a temporal reduction in pasture availability by spending more time grazing and less time ruminating. Similarly, the present experiment found that cows in the 7A treatment continued to graze at a time when (daylight) rumination peaked for cows in the 2A treatment. Secondly, ruminants have an intrinsic desire to graze herbage more intensively in the evening than in the morning, possibly to maximise intake when herbage is at its highest nutritive value [40,41,42] and provide a sufficient quantity of food for digestion during the night [41]. However, cows in the 7A treatment of this experiment were provided with ⅔ of their evening pasture allocation prior to nightfall, and the final ⅓ provided at approximately 0530 h the following morning. Fasted cattle have a progressively emptier rumen [4], and a low rumination time has been associated with less material in the rumen to digest (see [38]). Thus, during daylight hours the frequent presentation of fresh pasture may have stimulated grazing in 7A cows at the expense of rumination, while a low rumen fill could explain why 7A cows spent more time resting and less time ruminating overnight than 2A cows. Adoption of complex intensive strip-grazing regimes, with or without the assistance of virtual fencing technology [10,11], requires careful consideration of the temporal availability of pasture strips, the quantity of pasture available in each strip, and the interaction between these two factors. Understanding the effects of these factors on pasture dry matter intake may be even more important at higher levels of concentrate supplementation than those utilised in the present experiment. Indeed, the average milk response to supplementation declines with increasing rates of substitution of supplementary feed for pasture [43], making a more efficient use of supplements a key requirement to optimizing feed efficiency in pasture-based dairy systems [44].

Regardless of treatment, cows produced 3 L less milk in period 2 (i.e., days 20 to 28) than period 1 (i.e., days 3 to 14). This reduction in milk yield explains why SCC, expressed as a concentration of cells per mL, increased over time, and particularly for the 7A treatment. It also partially explains the effects of sample day on kilograms of protein and fat produced, although seasonal and paddock effects may have contributed. The rapid decline in milk yield reported in both treatments of the present research indicates the contribution of factors other than the natural reduction expected for dairy cows in mid-lactation. The *r*^2^ values for rising plate meter (RPM) calibration curves on irrigated perennial ryegrass at the same site as the present experiment were reported by Pembleton et al. [14] to range between 0.5 and 0.7. The RPM is a well-accepted and adopted tool for allocating pasture in pastoral dairy systems, and, when applied over a large herd, this inherent variability becomes marginal on a per cow basis. However, variation in RPM biomass estimations may have failed to achieve the precise pasture allocations required for the small herd sizes and tight allocations utilised by the present experiment, resulting in an inadvertent under-allocation of pasture. Nonetheless, based on DM estimations using a rising plate meter, cows in both treatments of the present study were allocated the same amount of feed and, although milk yield declined for all cows, significant effects of treatment on milk yield were reported.

An interesting finding of this research was that, although treatments were balanced for age and weight, older and heavier cows in the 7A treatment produced more milk, but the same relationships were not significant in the 2A treatment. This study placed considerable spatiotemporal restrictions on the availability of feed to 7A cows. Larger cows may have a competitive advantage under such conditions, partly because their increased mouth size which enables a higher intake per bite [45]. The space in which 7A cows could graze, and regulate social interactions around grazing, was also limited by the size of the intensive strip of pasture offered in the present research. For instance, the space allowance per strip for 7A cows ranged from 5.6 to 22.8 m^2^ per cow, whereas the minimum recommended space allowance for beef cattle in Australian feedlots is 9 m^2^ per cow [46]. In cattle, high social rank is associated with age [47,48], weight [47], and, when fed grain competitively, intake [37,47,49]. Stafford and Gregory [8] warned that increased intensification of grazing management may pose risks to the welfare of grazed cattle, particularly for animals of lower social status who could be forced away from fresh pasture [8,50]. The implications of intensification of dairy grazing systems for the most vulnerable animals in the herd is imperative to the development of ethical intensive grazing regimes. However, caution is required in interpreting these data, as they are based on correlational, rather than experimental and comparative, relationships for a small sample size (*n* = 30 per treatment).

## 5. Conclusions

Feeding mid-lactation dairy cows their daily pasture allowance over seven smaller grazing strips (7A cows) may have reduced the time cows spent ruminating, milk yield, and energy corrected milk yield, compared to cows offered fresh pasture twice per day (2A cows). However, milk composition, the time cows spent feeding, and pasture consumption do not appear to be affected by feeding frequency. Multiple brief periods of high intake rates may have led to poor mastication and improper packing of ingesta for 7A cows, resulting in reduced energy utilisation for productive purposes. Grazing may also have been stimulated at the expense of rumination in 7A cows, while a low rumen fill may have compromised rumination, particularly during the night. The success of increasingly intensive grazing regimes is dependent on a design that supports the natural ingestive, digestive, and social behaviours of cattle, rather than requiring cattle to substantially alter their behaviour in accordance with the grazing regime imposed upon them. Further research is needed to determine the details of complex intensive grazing regimes, including the effects that the temporal availability of pasture strips, the quantity of pasture available in each strip, and the interaction between these two factors, has on the behaviour and productivity of lactating dairy cows.

## Figures and Tables

**Table 1:** Summary of linear mixed models used to assess effects of feeding frequency on measures of dairy cow behaviour, milk production, live weight, and estimated pasture consumption ^1^.

Measure	Repeated Observations	Fixed Effects	Random Effects	Covariates
Behaviour				
per hour ^2^	Cow (period) over day	Treatment	Paddock	None
per day	Cow (period) over day	Treatment Prior treatment Treatment × prior treatment	Paddock	None
Milk composition	Cow (period) over day	Treatment Day (period) Treatment × day (period) Prior treatment	None	Milk composition at day −1
Live weight	Cow (period) over day	Treatment Day (period) Treatment × day (period) Prior treatment	None	Live weight at day −1
Milk yield	Cow (period) over day	Treatment Prior treatment Treatment × prior treatment	Paddock	Milk yield at day −1
Pasture consumption	Day [(paddock) period]	Treatment Period Treatment × period	None	Pre-grazed pasture biomass

^1^ Nesting within a higher order is indicated by parenthesis e.g., lower order (higher order). Treatment: feeding frequency of 2 or 7 allocations per day. Day: sample day (7, 14, 21, or 28) for milk composition and live weight; experimental day (1 to 28) for all other measures. Period: time period prior to (period 1: days 1–14) or following (period 2: days 15–28) treatment crossover. Prior treatment: In period 1 cows did not have a prior treatment (none). In period 2 “prior treatment” refers to the treatment the cow was in for period 1. ^2^ Rounded to the nearest minute and analysed using generalized linear mixed model (GLMM) with underlying Poisson distribution.

**Table 2:** Effect of frequency of provision of fresh pasture (Treatment: 2 allocations = 2 per day; 7 allocations = 7 per day) on cow live weight, milk composition, and the fat and protein corrected milk (FPCM) per sample day. Least square means and pooled standard errors are presented.

Variable	Treatment								SE_P_^1^	*p*-Value ^2^		
	2 Per Day				7 Per Day							
	Day 7	Day 14	Day 21	Day 28	Day 7	Day 14	Day 21	Day 28		T	P	D
Live weight (kg)	530 ^a^	523 ^b^	527 ^a^	518 ^b^	531 ^a^	521 ^b^	528 ^a^	521 ^b^	5.57	0.76	0.12	<0.001
Fat												
Percent	4.43	4.48	4.68	4.60	4.37	4.54	4.57	4.59	0.042	0.76	0.53	0.43
Total kg	1.02 ^ax^	0.84 ^ay^	0.99 ^ax^	0.87 ^ay^	0.95 ^bx^	0.84 ^by^	0.94 ^bx^	0.82 ^by^	0.011	0.05	0.29	<0.001
Protein												
Percent	3.22	3.24	3.26	3.32	3.23	3.26	3.29	3.29	0.024	0.86	0.58	0.39
Total kg	0.74 ^a^	0.60 ^b^	0.70 ^a^	0.63 ^b^	0.71 ^a^	0.60 ^b^	0.68 ^a^	0.59 ^b^	0.007	0.07	0.62	<0.001
SCC (×1000/mL) ^3^	36.1 ^a^	44.7 ^b^	61.7 ^c^	86.9 ^d^	49.1 ^a^	73.1 ^b^	101 ^c^	113 ^d^	34.4	0.09	0.39	<0.001
FPCM (kg)	23.9 ^ax^	19.6 ^ay^	22.8 ^ax^	20.2 ^ay^	22.5 ^bx^	19.5 ^by^	21.8 ^bx^	19.1 ^by^	0.214	0.03	0.39	<0.001

Within a row, means with different superscript letters ^a,b,c,d^ or ^x,y^ differ at *p* ≤ 0.05. ^1^ SE_P_ = pooled standard error. ^2^ T = Treatment; P = Prior Treatment; D = Sample Day. ^3^ y = Log_10_(X) transformed prior to analysis, back-transformed means presented.

**Table 3:** Linear mixed models examining the effects of feeding frequency (2 allocations = 2 feeds per day; 7 allocations = 7 feeds per day) on the time (h per cow and day) cows spent feeding, resting, and ruminating. Least square means and pooled standard errors (SE_P_) are presented.

Behaviour	Treatment		Prior Treatment ^1^			SE_P_	*p*-Value	
	2 Feeds	7 Feeds	Period 1	Period 2			Treatment	Prior Treatment
			None	2 Feeds	7 Feeds			
Feeding	8.9	8.9	8.8 ^a^	9.2 ^b^	8.9 ^ab^	0.19	0.95	0.047
Resting ^2^	5.6 ^a^	6.0 ^b^	5.6	5.57	5.93	0.79	0.008	0.18
Ruminating	7.7 ^a^	7.2 ^b^	8.0 ^a^	7.2 ^b^	7.0 ^b^	0.11	<0.001	<0.001

^a,b^ Within fixed effects (i.e., treatment or prior treatment) and rows, superscripts indicate where means differ. ^1^ In period 1 (days 1–14), cows did not have a prior treatment (none). In period 2 (days 15–28), “prior treatment” refers to the treatment the cow was in for period 1. ^2^ y = √X back-transformed means.

**Table 4:** Partial correlation coefficients (controlling for period, day, and paddock) between cow behaviour and variables relating to milk production and the cow, for cows fed pasture 2 or 7 times per day.

Cow Characteristic	2 Feeds Per Day			7 Feeds Per Day		
	Feeding	Ruminating	Resting ^1^	Feeding	Ruminating	Resting ^1^
Age	−0.07	−0.09 *	0.18 **	−0.13 **	0.06	0.16 **
Start weight	−0.07	−0.10 *	0.19 **	−0.04	−0.05	0.20 **
Days in milk (day −1)	−0.08	−0.04	0.02	−0.03	−0.01	0.06
Milk yield (L/day)	0.08 *	0.22 **	−0.16 **	−0.06	0.14 **	−0.003
FPCM (kg/day) ^2^	0.31 **	−0.06	0.05	0.07	−0.03	0.18

* Significant at *p* ≤ 0.05, ** significant at *p* ≤ 0.01; ^1^ y = √X transformed prior to analysis. ^2^ FPCM = Fat and protein corrected milk.
